# Pathogenesis, Diagnosis, and Management of Splenogonadal Fusion: A Literature Review

**DOI:** 10.1155/2020/8876219

**Published:** 2020-10-07

**Authors:** Youssef Kadouri, Damien Carnicelli, Hachem El Sayegh, Lounis Benslimane, Yassine Nouini

**Affiliations:** ^1^Faculty of Medicine and Pharmacy of Rabat Morocco, Ibn Sina Hospital, Department of Urology A, Mohammed V University, Morocco; ^2^Department of Urology and Andrology, Center Hospitalier Métropole Savoie, Chambery, France

## Abstract

**Introduction:**

Splenogonadal fusion is a rare congenital anomaly, defined by the presence of ectopic splenic tissue caused by an abnormal connection between the spleen and the gonad or mesonephrotic derivatives during the embryonic period.

**Materials and Methods:**

By reporting an observational case and performing a review of the literature according to the CARE guidelines (using the PubMed database and guidelines from urology, general surgery, and pediatric learned societies), we present the embryological genesis of the splenogonadal fusion, the associated anatomical anomalies, and the diagnostic procedure. *Observation*. We report the case of a patient aged 45, with no notable history, reporting left testicular pain. A small nodule on the upper pole of the left testicular was clinically palpable. Tumor markers were normal, and scrotal ultrasound depicted a hypoechoic hypervascular nodule measuring 8∗6∗8 mm. After validation in a multidisciplinary oncology consultation meeting and opinion from a uro-andrologist expert, the patient underwent an inguinal lumpectomy with an extemporaneous examination which did not objectify any signs of malignancy. Ultimately, it is a normal spleen tissue in the testicular ectopic position. *Discussion*. Splenogonadal fusion corresponds to a rare congenital malformation; less than 200 cases have been published in the literature, most often affecting boys, with a sex ratio of 15/1. Two types are described, depending on the continuity of the link between the orthotopic spleen and the gonad: the continuous and discontinuous forms. In a third of the cases, there are associated congenital malformations and particularly in the continuous forms (44 to 50% of the cases): anomalies of the limbs, micrognathia, microgyria, and hepatic and digestive abnormalities. Cryptorchidism is associated with the continuous form in 31% of cases. The preoperative diagnosis remains difficult because of its morphological and clinical characteristics suggesting a tumor process.

## 1. Introduction

Splenogonadal fusion (SGF) is a rare congenital malformation, defined by the presence of ectopic splenic tissue caused by an abnormal connection between the spleen and the gonad or mesonephrotic derivatives during the embryonic period. Described for the first time in 1883 by Farthouat et al. [1] The age of the patients is less than 10 years in half of the cases reported, and 82% of the cases occur in young men under the age of 30. [2] The sex ratio is 15/1 [3]. It is often associated with other congenital anomalies and poses a problem of differential diagnosis with testicular tumors.

## 2. Materials and Methods

By reporting an observational case and performing a review of the literature according to the CARE guidelines (using the PubMed database and guidelines from urology, general surgery, and pediatric learned societies), we present the embryological genesis of the splenogonadal fusion, associated anatomical anomalies, and the diagnostic procedure. We used the following key word associations in French and English: “splenogonadal fusion” (Fusion splénogonadique) AND “congenital anomalies” (Anomalies congénitales), “splenogonadal fusion” AND “cryptorchidism” (Cryptorchidie), “splenogonadal fusion” AND “testicular masses “, “ splenogonadal fusion” AND “limb defect syndrom”.

Only reviews published in English or French were analyzed. The reviews were selected on the basis of their level of evidence and their relevance.

## 3. Observation

**Figure 1:**
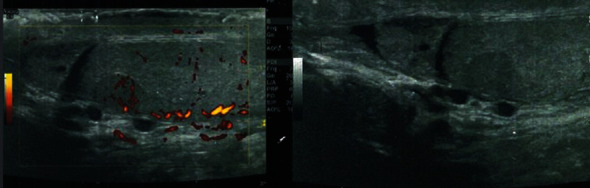
Scrotal ultrasound showing hypervascular hypoechogene testicular module measuring 8∗6∗8 mm.

We report the case of a 45-year-old patient, with no significant history, reporting left testicular pain for 5 years. A small solid nodule at the upper pole of the left testicular was clinically palpable. Scrotal ultrasound described a hypoechoic hypervascular nodule measuring 8∗6∗8 mm (Figure 1).

As a testicular tumor is strongly suspected, an assay for testicular tumor markers was requested: alpha-fetoprotein, human chorionic gonadotropin (hCG), and lactate dehydrogenase (LDH) were found to be normal.

**Figure 2:**
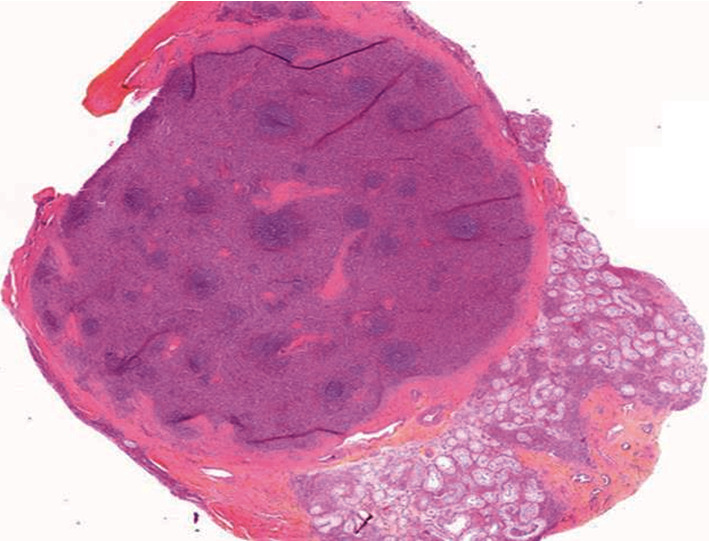
Microscopic examination: normal splenic tissue consisting of congestive red pulp and numerous mature lymphoid foci forming the white pulp, limited by a fibrous capsule.

Although the presentation of this file presents the key diagnostic elements of testicular cancer (hypoechogenic solid mass palpable intrascrotal with doppler hypervascularization), signs make this diagnosis suspect: the chronic pain felt by the patient, especially after intense physical exercise and negative tumor markers. After validation in a multidisciplinary consultation meeting and a uro-andrologist expert, the patient underwent an inguinal lumpectomy with an extemporaneous examination which did not objectify any signs of malignancy. Ultimately, the anatomopathological analysis concluded with normal splenic tissue in the testicular ectopic position (Figure 2).

## 4. Discussion

SGF is a rare benign congenital malformation, reported in detail by Carragher [4] in 1889, although Boestrom had described this anomaly in 1883. It was Sneath [5]. who published the first case of splenogonadic fusion in the American literature in 1913. Until 1917, all the reported cases were discovered fortuitously during autopsy studies, and thus, less than 200 cases were published in the literature [6]. This anomaly concerns almost exclusively the male sex; only 8 cases have been reported in women, [7] although this incidence is probably underestimated due to the inaccessibility of the ovaries to clinical examination. SGF mainly affects the left gonad between the fifth and sixth weeks of gestation (98% of cases) before the start of gonadal descent [8]. Note that a single case has been published in animals, more specifically in horses [9].

Putschar and Manion [10] describe two types of splenogonadal fusion: the continuous form characterized by the presence of a cord between the orthotopic spleen and the gonad and the discontinuous form which has no connection to the main spleen and the splenic tissue ectopic, directly fused to the testicular albuginea. Previous studies indicate that the frequency of the two types is equal [9], but other studies suggest that the frequency of the discontinuous type of SGF was lower than that of the continuous type [11, 12].

In the continuous form, the main spleen is connected to the gonad by an entirely fibrous cord or includes islets of splenic tissue, or even entirely composed of splenic tissue. [13] Most often, this cord comes from the upper pole of the spleen and ends at the upper pole of the gonad.

Congenital malformations occurring in the continuous form of SGF are 5 times more frequent than in the discontinuous form [12]. Cryptorchidism and inguinal hernias are the most frequently associated malformations. In 1980, Cortes et al. [14] examined 111 cases of splenogonadic fusion of which 31% had cryptorchidism (59% bilateral, 26% right intra-abdominal, and 65% left intra-abdominal). In case of continuous FSG, about 50% are accompanied by other congenital malformations, the most common of which are limb malformations: ectromelia, peromelia, amelia, phocomelia [15, 16], and micrognathia. [17] Other malformations are more exceptional: cardiac anomalies, [18] microgastronomy, [16] cleft palate, hypospadias, sexual ambiguities, varicocele, and spina bifida. [19] The association of splenogonadal fusion with major malformations of the limbs and mandible defines the splenogonadal fusion limb defect syndrome (SGFLD) of which 30 cases have been published in the literature; the SGFLD is marked by a perinatal mortality rate ranging from 40 to 50%. [1] Bonneau et al. [20] examined 29 cases of SGFLD, of which 24 cases (82.7%) were continuous, while 70% were associated with micrognathia.

The discontinuous forms are quite different since there are generally no associated malformations. The gonad is fused with a supernumerary spleen with no connection to the main spleen. The splenic tissue can be simply adherent to the elements of the cord or can be enveloped by the albugine tunic of the testicle, but it can also be separated from it; in this form, the testicle is often ectopic [21, 22], sometimes dysgenic, explaining possible degeneration [21].

Our case was of the discontinuous type, as no link between the main spleen and the testicle was objectified, either by ultrasound or during surgical exploration, and the patient was free from any associated clinically evident malformation and the clinical examination did not objectify limb or mandible malformations, which agrees with the data in the literature.

**Figure 3:**
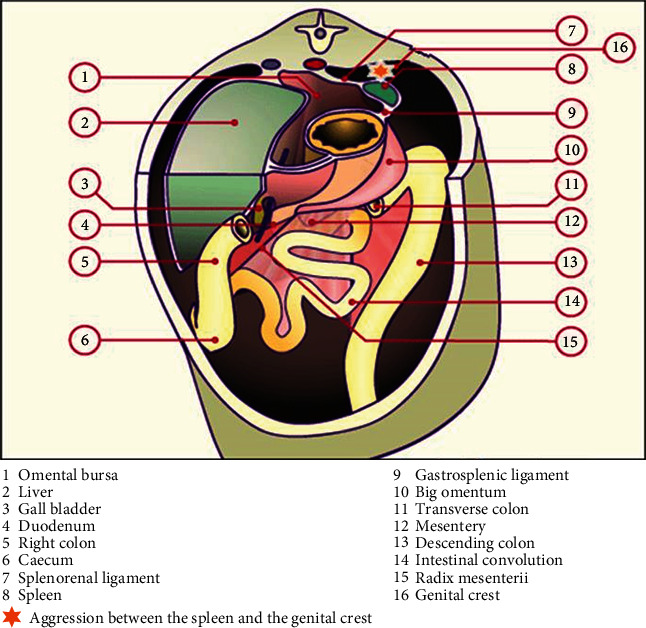
Horizontal section at the height of the stomach and spleen explaining the SGF genesis after gastric rotation and the splenic draft of the genital crest between the 5th and 8th weeks of gestation.

**Figure 4:**
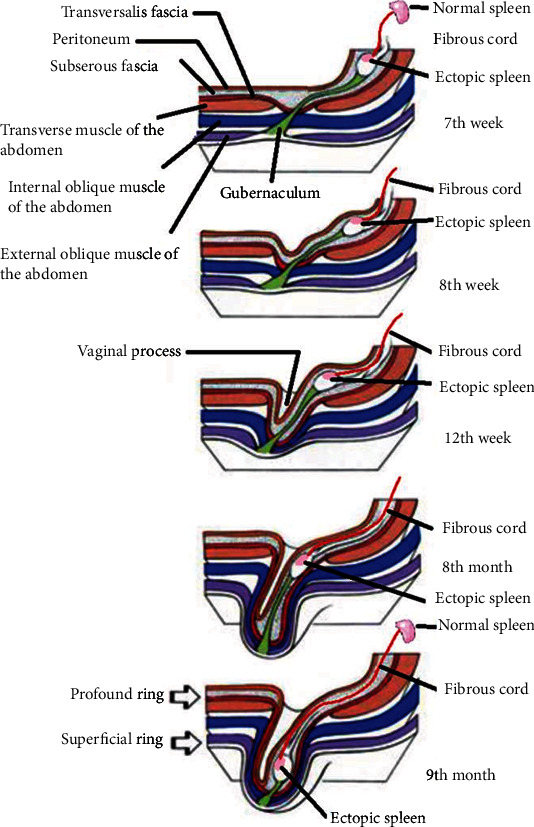
Diagram explaining the genesis of the continuous form of the SGF during the gonadal descent.

The exact pathogenesis of splenogonadic fusion is not yet clear, but it is generally believed to occur before gonadal descent, between the fifth and sixth weeks of pregnancy. During embryogenesis and precisely from the fifth week of gestation, the stomach is moved to the left of the median plane and turns around its axis, this gastric rotation brings together the two spleen and gonad tissues, an event occurring during this period can cause fusion of the surface of the developing genital ridge and the splenic outline (Figure 3), which accounts for the frequency of left localization of splenogonadal fusions, although cases of fusion of splenic tissue with the right testicle have been described. Some authors also evoke the possibility of migration of spleen cells by retroperitoneal route under the effect of an unknown teratogenic agent, [23] or of an hereditary participation in an autosomal recessive form. [24] The subsequent descent of the gonad between the 8th and the 10th week leads to a simultaneous descent of part of the spleen (Figure 4), so that the spleen tissue can appear in any place of the gonadal descent path, even in the inguinal canal or in the scrotum to arrive at the continuous form of SGF [12, 19]. These embryological hypotheses allow us to underline the importance of the classification proposed by Guarin et al., [15] distinguishing two types of SGF with quite different consequences (Table 1).

The clinical presentation of SGF is not specific, and the diagnosis is often accidentally made during surgery for inguinal hernia and/or cryptorchidism, which are the two most frequently associated anomalies or on histological examination after an orchidectomy for tumor. The discontinuous form usually presents as a hard scrotal nodule, imitating a testicular tumor, often asymptomatic, unless the patient develops a disease with splenic involvement (leukemia, mononucleosis, malaria, and salmonellosis [14]), because the splenic ectopic tissue is also affected, causing symptoms of increased volume and pain. Its association with germ tumors has been described in a few cases with the particularity that these patients had ipsilateral cryptorchidism. Other cases have been discovered in front of intestinal obstruction caused by the intraperitoneal cord [25], traumatic rupture of the ectopic spleen, or an association with an intra-abdominal seminoma. However, some rare cases have also been diagnosed preoperatively by ultrasound, computed tomography, or magnetic resonance imaging, [26, 27] most were of the continuous type, demonstrating a tubular structure fused with the testes. Splenic scintigraphy using technetium-99 m (99mTc) is a valid option once splenogonadic fusion is suspected. The binding of the radioactive tracer in the spleen and testicular mass is similar, confirming the ectopic splenic origin of this mass. [28]

**Figure 5:**
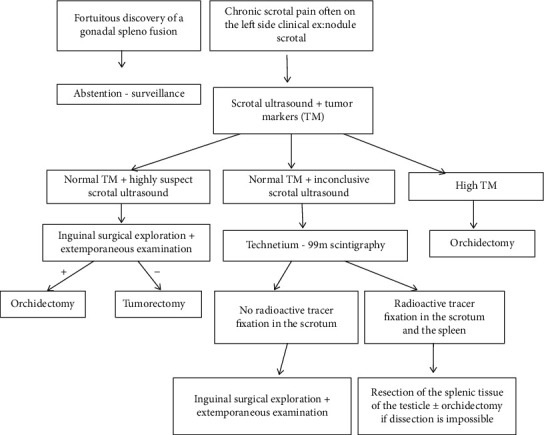
Decision tree in front of a chronic testicular nodule.

Given this clinical diversity and diagnostic difficulty, we propose a diagnostic algorithm for each chronic testicular nodule (Figure 5).

The treatment of splenogonadic fusion is controversial, even if it is an almost always mild pathology. For some authors, surgery is compulsory in order to confirm the diagnosis and exclude the infrequent association with a testicular neoplasm [11]. The association with a testicular tumor has only been described in four cases [10]. There is no obvious causality between the splenogonadic fusion and the malignant transformation. The rare cases observed were probably prone to developing testicular neoplasm due to cryptorchidism [29]. If surgery is performed, orchidectomy is generally not indicated, splenic tissue can usually be easily dissected from gonadal structures and the testicle can be kept. [4, 30] For other authors, abstention can be discussed if the anomaly is recognized preoperatively and if it does not manifest clinically. [31]

## 5. Conclusion

Splenogonadal fusion is a rare benign congenital disease with diagnostic difficulties due to the absence of typical clinical symptoms. Better knowledge of this disease will prevent a misdiagnosis of testicular tumors and the need for an unnecessary orchidectomy. We offer a decision tree for a better management of this pathology.

## Figures and Tables

**Table 1:** Comparison between the continuous and discontinuous form of the SGF.

	SGF continuous type	SGF discontinuous type
Definition	There is a cord between the main spleen and the gonad	Lack of connection between the main spleen and the gonad or ectopic splenic tissue
Frequency	56%	44%
Associated malformations	Common: cryptorchidism	Uncommon
Imaging (ultrasound, CT, MRI)	Visualization of the connecting cord between the main spleen and the gonad	No link between the main spleen and the gonad
Tc-99 m scintigraphy	Similar fixation of the radioactive tracer in the main spleen and suspect mass	Similar fixation of the radioactive tracer in the main spleen and suspect mass

## Data Availability

By reporting an observational case and performing a review of the literature according to the PRISMA recommendations (using the PubMed database and guidelines from urology, general surgery and pediatrics learned societies), we present the embryological genesis of the splenogonadal fusion, associated anatomical anomalies, and the diagnostic procedure. We used the following key word associations in French and English: “splenogonadal fusion” (Fusion splénogonadique) AND “congenital anomalies” (Anomalies congénitales), “splenogonadal fusion” AND “cryptorchidism” (Cryptorchidie), “splenogonadal fusion” AND “testicular masses ", “splenogonadal fusion” AND “limb defect syndrom”

## References

[1] Farthouat P., Faucompret S., Debourdeau P., Cruel T., Breda Y. (2001). Unusual testicular tumor: an ectopic spleen. *Annales de chirurgie*.

[2] Khairat A. B. M., Ismail A. M. (2005). Splenogonadal fusion: case presentation and literature review. *Journal of Pediatric Surgery*.

[3] Moore P. J., Hawkins E. P., Galliani C. A., Guerry-Force M. L. (1997). Splenogonadal fusion with limb deficiency and micrognathia. *Southern Medical Journal*.

[4] Carragher A. M. (1990). One hundred years of splenogonadal fusion. *Urology*.

[5] Sneath W. A. (1913). An apparent third testicle consisting of a scrotal spleen. *Journal of Anatomy and Physiology*.

[6] Jayasundara J. A. S. B., Vithana V. H., Lamahewage A. K. (2013). A case of continuous-type splenogonadal fusion. *Singapore Medical Journal*.

[7] Meneses M. F., Ostrowski M. L. (1989). Female splenic-gonadal fusion of the discontinuous type. *Human Pathology*.

[8] Gouw A. S., Elema J. D., Bink-Boelkens M. T., de Jongh H. J., ten Kate L. P. (1985). The spectrum of splenogonadal fusion. Case report and review of 84 reported cases. *European Journal of Pediatrics*.

[9] Noakes D. E., White R. A. (1976). Splenic-gonadal fusion in the horse. *The Veterinary Record*.

[10] Putschar W. G., Manion W. C. (1956). Splenicgonadal fusion. *The American Journal of Pathology*.

[11] Lopes R. I., Medeiros M. T. ., Arap M. A., Cocuzza M., Srougi M., Hallak J. (2012). Splenogonadal fusion and testicular cancer: case report and review of the literature. *Einstein (Sao Paulo)*.

[12] Bosnalı O., Cici İ., Moralıoğlu S., Cerrah-Celayir A. (2014). Continuous-type splenogonadal fusion: report of a rare case. *The Turkish Journal of Pediatrics*.

[13] Lungarella G., Fonzi L., De Martino A., Giacchi M. (1980). A rare case of continuous type spleno-gonadal fusion. *Annales d'anatomie pathologique*.

[14] Cortes D., Thorup J. M., Visfeldt J. (1996). The pathogenesis of cryptorchidism and splenogonadal fusion: a new hypothesis. *British Journal of Urology*.

[15] Guarin U., Dimitrieva Z., Ashley S. J. (1975). Splenogonadal fusion-a rare congenital anomaly demonstrated by 99Tc-sulfur colloid imaging: case report. *Journal of nuclear medicine: official publication, Society of Nuclear Medicine*.

[16] Mandell G. A., Heyman S., Alavi A., Ziegler M. M. (1983). A case of microgastria in association with splenic-gonadal fusion. *Pediatric Radiology*.

[17] Tsingoglou S., Wilkinson A. W. (1976). Splenogonadal fusion. *The British Journal of Surgery*.

[18] Loomis K. F., Moore G. W., Hutchins G. M. (1982). Unusual cardiac malformations in splenogonadal fusion-peromelia syndrome: relationship to normal development. *Teratology*.

[19] Le Roux P. J., Heddle R. M. (2000). Splenogonadal fusion: is the accepted classification system accurate?. *BJU International*.

[20] Bonneau D., Roume J., Gonzalez M. (1999). Splenogonadal fusion limb defect syndrome: report of five new cases and review. *American Journal of Medical Genetics*.

[21] Falkowski W. S., Carter M. F. (1980). Splenogonadal fusion associated with an anaplastic seminoma. *The Journal of Urology*.

[22] Finkbeiner A. E., DeRidder P. A., Ryden S. E. (1977). Splenic-gonadal fusion and adrenal cortical rest associated with bilateral cryptorchism. *Urology*.

[23] Cirillo R. L., Coley B. D., Binkovitz L. A., Jayanthi R. V. (1999). Sonographic findings in splenogonadal fusion. *Pediatric Radiology*.

[24] Balaji K. C., Caldamone A. A., Rabinowitz R., Ortenberg J., Diamond D. A. (1996). Splenogonadal fusion. *The Journal of Urology*.

[25] Hines J. R., Eggum P. R. (1961). Splenic-gonadal fusion causing bowel obstruction. *Archives of Surgery*.

[26] Varma D. R., Sirineni G. R., Rao M. V., Pottala K. M., Mallipudi B. V. P. (2007). Sonographic and CT features of splenogonadal fusion. *Pediatric Radiology*.

[27] Croxford W. C., Pfistermuller K. L. M., Scott F., Pope A. J. (2015). Splenogonadal fusion presenting clinically and radiologically as a seminoma. *Urology case reports*.

[28] Ando S., Shimazui T., Hattori K., Yamamoto T., Kuriyagawa K., Akaza H. (2006). Splenogonadal fusion: case report and review of published works. *International Journal of Urology*.

[29] Sountoulides P., Neri F., Bellocci R., Schips L., Cindolo L. (2014). Splenogonadal fusion mimicking a testis tumor. *Journal of Postgraduate Medicine*.

[30] Verga G., Parigi G. B. (1990). Splenic gonadal fusion: a plea for conservative surgery. *Urology*.

[31] Karaman M. I., Gonzales E. T. (1996). Splenogonadal fusion: report of 2 cases and review of the literature. *The Journal of Urology*.

